# Robotic Excision of a Canal of Nuck Cyst During Bilateral Inguinal and Left Femoral Hernia Repair: A Case Report

**DOI:** 10.7759/cureus.104375

**Published:** 2026-02-27

**Authors:** Jorge A Dorantes, Dimitri Tchienga, Raema Jean, Michelle Gallas, Shohab Virk, Jorge R Rabaza, Khaled Y Omar, Anthony Gonzalez

**Affiliations:** 1 Department of General Surgery, Baptist Health South Florida, Miami, USA; 2 Center for Research, Baptist Health South Florida, Miami, USA; 3 Department of General Surgery, Baptist Hospital of Miami, Miami, USA; 4 Department of Surgery, Baptist Health South Florida, Miami, USA

**Keywords:** bilateral inguinal hernia, canal of nuck hydrocele, femoral hernia, robotic surgery, transabdominal preperitoneal (tapp)

## Abstract

Robotic surgery has become the preferred approach in many surgical procedures due to advantages involving reduced blood loss, shorter hospital stay, faster recovery time, and enhanced dexterity. Here, we describe the case of a 62-year-old woman with an unremarkable past medical history who presented with painful swelling in the right inguinal region for two months. Ultrasonography revealed a well-circumscribed, fluid-filled ovoid structure with a small narrow neck extending superiorly within the right inguinal canal, consistent with a canal of Nuck cyst. This case report describes, to the best of our knowledge, the first preoperatively diagnosed canal of Nuck cyst and concurrent bilateral inguinal and femoral hernia repair resected robotically and highlights the feasibility of adopting a robotic versus a laparoscopic approach.

## Introduction

In the 17th century, the processus vaginalis peritonei was described as the canal of Nuck, named after the Dutch anatomist Anton Nuck; it is located within the inguinal canal of the female reproductive system [1].

Anatomically, the canal of Nuck is a small evagination attached to the round ligament of the uterus and extending through the superficial inguinal ring into the inguinal canal in female infants [1]. In women, the canal of Nuck typically obliterates within the first year of life [1]. A cyst may develop when the distal portion of the canal fails to obliterate [1].

Three types of canal of Nuck cysts have been described. Type I (most common) has no communication with the peritoneal cavity and may occur anywhere along the round ligament from the internal ring to the labia majora [1,2]. Type II is analogous to a congenital hydrocele in men, in which communication with the peritoneal cavity remains patent [1,2]. Type III (hourglass type) involves constriction at the deep ring, with a proximal preperitoneal component and a distal component in the inguinal canal, which may clinically simulate a hernia [1,2]. The patient reported here was found to have a type I cyst.

Compared with the male equivalent (spermatic cord hydrocele), this entity is rare and less familiar to clinicians. Diagnosis relies on clinical presentation, physical examination findings, and radiographic imaging. Although no standardized treatment exists, open and laparoscopic approaches have been reported with successful outcomes [1,2]. Both techniques can be limited by visualization, whereas robotic surgery may enhance surgical precision via its magnified 3D imaging capabilities [3,4]. In this report, we describe the robotic excision of a canal of Nuck cyst with concurrent inguinal hernia repair.

## Case presentation

**Figure 1:**
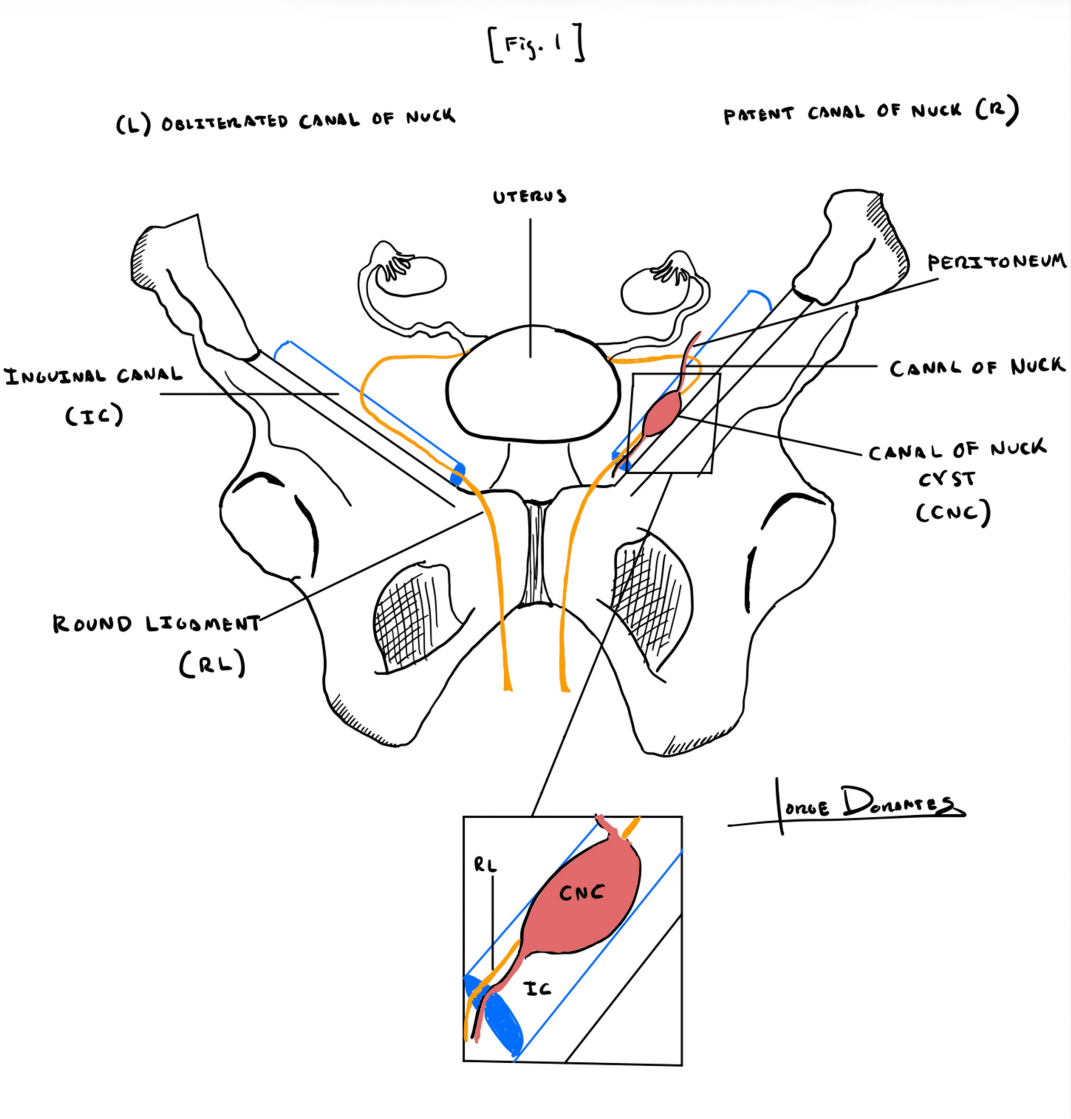
Canal of Nuck Anatomy The canal of Nuck is a patent pouch of parietal peritoneum that extends from the round ligament, passes through the inguinal canal, and terminates at the labia majora. Here, the female lower abdominal wall is depicted. Illustration created by Jorge A. Dorantes, MD, ©2026

**Figure 2:**
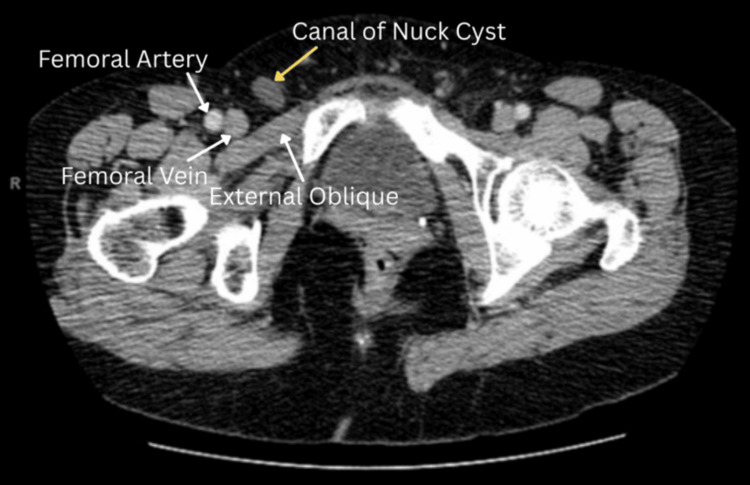
CT of the Abdomen and Pelvis With an Intravenous Contrast of a Canal of Nuck Cyst A CT of the abdomen and pelvis with contrast shows a canal of Nuck cyst (yellow arrow), identified as a non-enhancing, well-circumscribed ovoid mass CT: computed tomography

**Figure 3:**
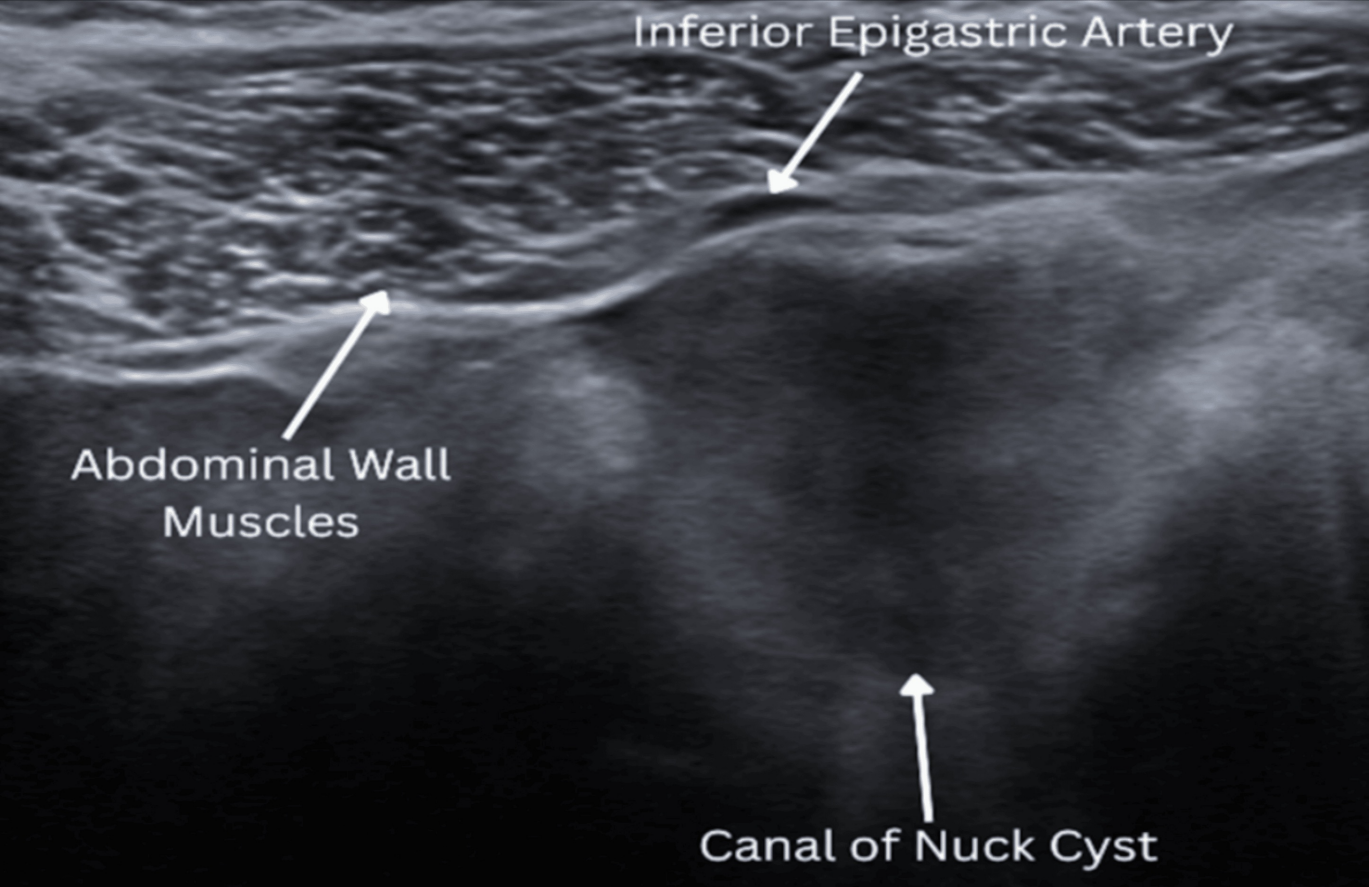
Ultrasound of a Canal of Nuck Cyst Ultrasound shows a 4.2 × 4.4 cm ill-circumscribed, fluid-filled structure in the right inguinal canal, representing a canal of Nuck cyst

A 62-year-old woman presented to the surgeon’s office with complaints of painful swelling in her right inguinal region for two months. The pain was moderate, non-radiating, aggravated by heavy lifting, and alleviated with rest. Physical examination revealed a right groin mass. Of note, a cyst (illustrated anatomically in Figure 1) was incidentally found on a computed tomography (CT) (Figure 2) in 2017 but was not symptomatic until two years later (2019). Ultrasonography performed on the patient in 2019 (Figure 3) demonstrated a well-circumscribed, fluid-filled ovoid structure measuring 1.2 cm × 4.2 cm × 4.4 cm with a small narrow neck extending superiorly within the right inguinal canal, consistent with a canal of Nuck cyst. Further diagnosis showed no change with Valsalva and no herniation of bowel loops. She underwent a robotic excision of the cyst with concomitant bilateral inguinal and left femoral hernia repair with mesh placement. The patient was discharged home a few hours after surgery with no complications and was subsequently evaluated in a postoperative follow-up at one week, showing good recovery.

**Figure 4:**
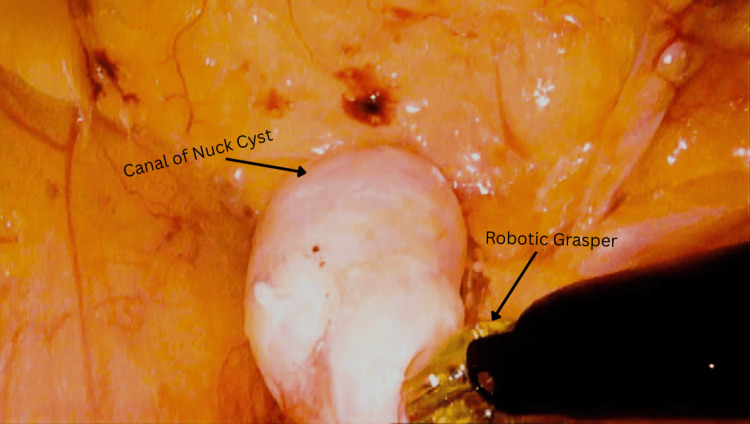
Intraoperative Image of the Right Inguinal Hernia Containing a Canal of Nuck Cyst A canal of Nuck cyst is described as a well-circumscribed, fluid-filled ovoid structure. Here, it is depicted measuring 1.2 cm × 4.2 cm × 4.4 cm. The robotic grasper (black object at the bottom right of the image) serves as one of the instruments used to reduce the cyst in the abdominal cavity

**Figure 5:**
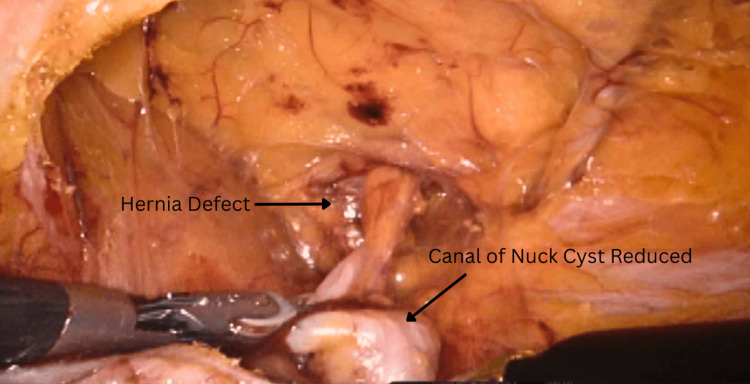
Intraoperative View of the Right Inguinal Hernia Defect (Top), Along With a Reduced Canal of Nuck Cyst (Bottom) The image depicts a complete reduction of the canal of Nuck cyst that was trapped in the right inguinal canal defect

After the administration of general anesthesia, the patient was prepped and draped. Three incisions were made 15 cm superior to the pubic tubercle, equally spaced across the abdomen. After the successful and safe establishment of pneumoperitoneum, the DaVinci XI (Intuitive Surgical, Sunnyvale, CA) was docked, and trocars were placed under direct visualization. The peritoneal flap was created at least 6 cm above the internal inguinal ring. The round ligament was transected with bipolar cautery. The hernial sac and canal of Nuck cyst were reduced in their entirety (Figures 4, 5). The cyst was resected and removed from the abdomen via an Endo Bag (Medtronic, Minneapolis, MN). Next, similar steps were used to reduce hernia contents in the left inguinal and femoral orifices. The complete dissection of the preperitoneal inguinal space was performed from the pubic tubercle, below Cooper’s ligament, and along the lateral abdominal wall, prior to the placement of mesh. Two ProGrip meshes (Medtronic, Minneapolis, MN) were used in this case per the surgeon’s preference; they were unfolded and applied to the bilateral myopectineal orifice. The peritoneum was closed with a 3-0 absorbable barbed suture. Skin incisions were closed with subcuticular suture and Dermabond (Ethicon, Raritan, NJ). The patient was evaluated in a postoperative follow-up at one week and at five months, showing good recovery.

## Discussion

The etiology of canal of Nuck cyst formation remains uncertain. Proposed mechanisms include a dysregulation of fluid movements of the processus vaginalis [1].

Imaging modalities used to evaluate the canal of Nuck abnormalities include ultrasound (US), computed tomography (CT), and magnetic resonance imaging (MRI). On US, the cyst may appear hypoechoic, while CT may show a thin-walled, non-enhancing cystic lesion [5]. The differential diagnosis includes Bartholin gland cyst, lymphadenitis, inguinal hernia, leiomyoma, and (when symptoms fluctuate with menses) endometriosis [3,5]. In this case, the US confirmed a canal of Nuck cyst, and an incidental CT performed two years later demonstrated a well-circumscribed cystic structure without evidence of compromised blood flow.

The surgical management of canal of Nuck cysts associated with inguinal hernia is recommended to prevent complications such as incarceration, infection, and bowel incarceration with ischemia. Open repair has historically been the dominant approach, but laparoscopic repair gained adoption due to reduced postoperative pain, earlier recovery, and shorter hospital stays [6]. Robotic surgery has more recently emerged as an alternative approach, with reported feasibility and safety and with the potential advantage of the improved visualization of both the cyst and hernia [3]. Comparative studies of robotic versus laparoscopic inguinal hernia repair suggest similar or lower postoperative pain in the robotic group [7].

Although robotic management appears feasible and safe with minimal complications, type III canal of Nuck cysts may be technically more challenging with minimally invasive approaches [8]. Surgeons should therefore evaluate risks carefully when selecting between transabdominal preperitoneal (TAPP), total extraperitoneal (TEP), or open Lichtenstein repair, with conversion to an open approach as an option when minimally invasive cases become complex intraoperatively [9].

Among minimally invasive techniques, TAPP cystectomy with mesh placement is most commonly reported [3]. Mesh-based hernioplasty is associated with lower long-term recurrence rates and supports routine reinforcement when appropriate [10]. In minimally invasive hernioplasty, the ligation of the round ligament may be performed when it obstructs reduction or visualization [3,11]. In our case, the round ligament was ligated to optimize exposure. Large cysts or hernias may widen the internal inguinal ring, which can be effectively addressed with mesh reinforcement [11].

Published outcomes following the laparoscopic resection of canal of Nuck cysts with inguinal hernia repair are favorable [12-20]. Across reports with follow-up ranging from three to six months, complications are typically minor (most commonly seroma or hematoma) and managed conservatively, with low reported recurrence during the same follow-up interval [12-20]. In our case, follow-up at one week demonstrated no complications or recurrence.

## Conclusions

A canal of Nuck cyst must always be included in the differential diagnosis of inguinal swelling in women. Although rare and difficult to identify, performing a good clinical history, conducting a thorough physical examination, and utilizing various imaging modalities all equally contribute to arriving at a proper diagnosis. Though open resection and possible inguinal and femoral hernia repair may be appropriate, the use of minimally invasive techniques, specifically robotic surgery, may be beneficial for the patient. Mesh utilization is indicated and recommended for the management of certain canal of Nuck cysts and concurrent inguinal and femoral hernia.
